# Low temperature response index for monitoring freezing injury of tea plant

**DOI:** 10.3389/fpls.2023.1096490

**Published:** 2023-02-02

**Authors:** Yilin Mao, He Li, Yu Wang, Kai Fan, Jiazhi Shen, Jie Zhang, Xiao Han, Yujie Song, Caihong Bi, Litao Sun, Zhaotang Ding

**Affiliations:** ^1^Tea Research Institute, Qingdao Agricultural University, Qingdao, China; ^2^Tea Research Institute, Shandong Academy of Agricultural Sciences, Jinan, China; ^3^Agricultural Technology Extension Center, Linyi Agricultural and Rural Bureau, Linyi, China

**Keywords:** tea plants, cold damage assessment, hyperspectral imaging, deep learning, LTRI

## Abstract

Freezing damage has been a common natural disaster for tea plantations. Quantitative detection of low temperature stress is significant for evaluating the degree of freezing injury to tea plants. Traditionally, the determination of physicochemical parameters of tea leaves and the investigation of freezing damage phenotype are the main approaches to detect the low temperature stress. However, these methods are time-consuming and laborious. In this study, different low temperature treatments were carried out on tea plants. The low temperature response index (LTRI) was established by measuring seven low temperature-induced components of tea leaves. The hyperspectral data of tea leaves was obtained by hyperspectral imaging and the feature bands were screened by successive projections algorithm (SPA), competitive adaptive reweighted sampling (CARS) and uninformative variable elimination (UVE). The LTRI and seven indexes of tea plant were modeled by partial least squares (PLS), support vector machine (SVM), random forests (RF), back propagation (BP) machine learning methods and convolutional neural networks (CNN), long short-term memory (LSTM) deep learning methods. The results indicated that: (1) the best prediction model for the seven indicators was LTRI-UVE-CNN (R^2 =^ 0.890, RMSEP=0.325, RPD=2.904); (2) the feature bands screened by UVE algorithm were more abundant, and the later modeling effect was better than CARS and SPA algorithm; (3) comparing the effects of the six modeling algorithms, the overall modeling effect of the CNN model was better than other models. It can be concluded that out of all the combined models in this paper, the LTRI-UVE-CNN was a promising model for predicting the degree of low temperature stress in tea plants.

## 1 Introduction

Tea plant (*Camellia sinensis* (L.) O. Kuntze.) is an evergreen crop, which thrives in warm temperatures and is sensitive to low temperatures (Li et al., 2011). In the context of climate change, low temperature has become one of the major environmental factors affecting the overwintering of tea plants and the growth of spring tea, causing economic loss of tea production. Therefore, rapid prediction of freezing injury of tea plants is a key issue to reduce the impact of freezing damage on tea production. Tea plants was provoked massive physiologic and metabolic reprogramming to adapt to the decline of ambient temperature, thereby enhancing the low temperature tolerance. For instance, the increase of soluble sugar (SS) in tea leaves suffered from cold injury can maintain the osmotic balance of cells, reduce the low temperature damage, and enhance the cold resistance (Ban et al., 2017). Suffering from unfavorable conditions, the biosynthesis of chlorophyll was blocked, resulting in the decrease of chlorophyll content in tea leaves (Liu et al., 2019a). In addition, the activity of antioxidant enzymes can be used to assess low temperature sensitivity or tolerance of plants. Catalase (CAT), peroxidase (POD) and superoxide dismutase (SOD) can eliminate the accumulated reactive oxygen induced by low temperature stress, thereby protecting cells from injury (Wang et al., 2021). Malondialdehyde (MDA) is a peroxidation product produced by cell membrane damage under low temperature stress, and its content is an important indicator to measure whether the cytoplasmic membrane damaged is serious (Liu et al., 2012).

Traditionally, the determination of physicochemical parameters of tea leaves and the investigation of freezing damage phenotype are the main approaches to detect the low temperature stress. However, these methods have distinct deficiency such as strong destructiveness, time-consuming and labor-intensive, low accuracy and lagging prevention. Therefore, there is an urgent need to propose a non-destructive, rapid and accurate monitoring method for low temperature-induced components.

Hyperspectral imaging (HSI) is an emerging optical technology. It has emerged as an economical alternative to traditional destructive sampling and laboratory testing. HSI combines the advantages of traditional imaging and spectroscopic techniques, in which, the surface structure information and the internal feature information of the object to be measured can be acquired simultaneously. This technology has been widely used for the non-destructive and rapid detection of chemical components in wheat (Duan et al., 2019), sugar beet (Pan et al., 2016), red jujube (Li et al., 2022c), strawberry (Weng et al., 2020) and other crops. In addition, HSI is also used in the compositional analysis of fresh or dried tea leaves. For example, Sonobe et al (Sonobe et al., 2018). used HSI to obtain spectral images of tea planted under low light stress, and combined model inversion and machine learning algorithms to quantify the chlorophyll content in tea leaves. Wang et al (Wang et al., 2018)identified nitrogen levels in tea plants based on hyperspectral imaging technique combined with support vector machine (SVM). Chen et al (Chen et al., 2021). used HSI combined with machine learning algorithm to predict the contents of MDA and SS in tea leaves under drought stress. However, there are few studies on HSI-based monitoring of cold stress-induced components in tea plants. At present, the research on hyperspectral detection of low temperature stress was mainly focused on wheat, and good results have been achieved (Wang et al., 2016; Feng et al., 2018). This provided a reference for the rapid and non-destructive detection of low temperature stress in tea plants. Therefore, HSI can provide new means and ideas for the rapid detection of low temperature-induced components and the evaluation of freezing injury degree in tea plants.

In order to help researchers faster calculation speed and computational robustness of the model, feature selection algorithms and machine learning algorithms were integrated in the practical application of hyperspectral data. There is redundant information in hyperspectral images that are irrelevant to the research goal. Therefore, it is particularly important to determine which information contributes more to the research objective (Su et al., 2021). In order to reduce dimensionality and remove redundant spectra, band selection methods such as successive projections algorithm (SPA), competitive adaptive reweighted sampling (CARS), and uninformative variable elimination (UVE) have been utilized by many researchers (Li et al., 2016b; Liu et al., 2019b). Currently, various machine learning methods have been used to build models based on spectral images (Li et al., 2022b). For example, Chen et al (Chen et al., 2022). used partial least squares (PLS), SVM and random forests (RF) models to model Na in crop leaves and compared their predictive performance. Moreover, as a research hotspot of machine learning, deep learning algorithms can achieve accurate feature extraction and have been used for the analysis of hyperspectral images (Qureshi et al., 2017). Recently, Zhang et al (Zhang et al., 2022). established a monitoring model of corn water content based on hyperspectral images by PLS, convolutional neural networks (CNN), long short-term memory (LSTM) and CNN-LSTM. However, few studies combined HSI with machine learning and deep learning methods to monitor the key components of tea plants under low temperature stress.

In this study, tea leaves with different freezing damage degrees were collected, the biochemical components and hyperspectral images of the samples were obtained. Multivariate scattering correction (MSC), Savitzky-Golay (S-G) and first derivative (1stD) algorithms were used to preprocess the spectral data; SPA, CARS and UVE algorithms were used to screen the characteristic bands of spectral data; SVM, RF, PLS, back propagation (BP) machine learning and CNN, LSTM deep learning algorithms were integrated. The prediction models for SPAD, SS, MDA contents, and CAT, POD, SOD activities of tea leaves were established, and the frost damage of tea plants was evaluated. The main focuses of this study are (1) develop a low temperature response index (LTRI) to comprehensively evaluate the freezing damage degree of tea plants; (2) explore multiple combinatorial models to estimate the performance of key low temperature-induced components and LTRI in tea leaves; (3) compare the optimization effects of SPA, CARS and UVE screening feature band methods on the prediction model; (4) discuss the prediction capacity of deep learning and traditional machine learning models.

## 2 Materials and methods

### 2.1 Experimental design

This experiment was carried out in the Tea Biology Laboratory of Qingdao Agricultural University from August to September 2021. The test materials were 2-year-old “Zhongcha 108” and “Longjing 43” tea plants. On August 20, 2021, the substrate (PINDSTRUP, Denmark) was put into a plastic nursery pot (8*8cm), 300 tea plants of each variety were planted, and then placed in an artificial climate box (RDN-1000D-4, 0~50°C, Ningbo Southeast Instrument Co., Ltd.) for pre-culture. Conditions in the climate box were as follows: temperature, 25°C/20 °C (16 h light/8 h dark) and light intensity, 10000 Lx. The tea plants were continuously cultured for 10 days. On August 31, 2021, according to the temperature treatment and varieties, the tea plants were moved into an artificial climate box (RXZ-0450, -10~50°C, Ningbo Jiangnan Instrument Factory) in batches for testing. The detailed treatment settings of low temperature stress are shown in **Table 1**. In order to reduce the experimental error, 6 times of low temperature treatment tests were conducted for each variety. 16 plants were randomly selected for each cold treatment, and two mature leaves were picked from each tea plant as a sample. Then, the harvested samples were subjected to hyperspectral data acquisition and chlorophyll content (SPAD value) determination, and then quickly frozen in liquid nitrogen and stored at -80°C until the physiological and biochemical indicators were determined. In this experiment, a total of 192 samples were collected.

**Table 1:** Treatment settings of low temperature stress.

Representations	Treatments	
	Temperature (°C)	Time (hours)
Control Check 8 (CK8)	25°C	8
Control Check 12 (CK12)		12
Chilling 8 (C8)	4°C	8
Chilling 12 (C12)		12
Freezing 8 (F8)	-4°C	8
Freezing 12 (F12)		12

### 2.2 Data acquisition

#### 2.2.1 Determination of physicochemical parameters and establishment of LTRI

Before the samples were cryopreserved, the chlorophyll content of each leaf was measured by a portable chlorophyll meter (SPAD-502, Japan). Each leaf was measured at 6 different positions and leaf veins were avoided manually. The SPAD value of tea leaves was the mean value of the 6 measured points.

Tea leaves were frozen by liquid nitrogen and ground into powder. The contents of SS and MDA and the activities of protective enzymes CAT, SOD and POD were determined according to the kit of Suzhou Grace Biotechnology Co., Ltd. SS, MDA content and protective enzyme CAT, SOD, POD activity kits are all microplate method, and the product numbers are G0501W, G0109W, G0105W, G0103W and G0107W respectively.

The establishment of LTRI. To quantitatively and comprehensively analyze the low temperature stress of tea leaves, LTRI was constructed. Firstly, the 6 major physiological indexes (i.e., SPAD, SS, MDA, CAT, POD, SOD) related to low temperature stress were standardized and analyzed by SPSS 25.0 software. Then, the linear combination coefficient (LCC) and the comprehensive score coefficient (CSC) were calculated by the formulas (1) and (2) respectively, and the LTRI was finally obtained.


(1) $$LCC_{ij}=LC_{ij}/\sqrt{\lambda_{j}}$$

(2) $$CSC_{i}=\sum_{i=1}^{6}\left(LCC_{i}\times VC_{i}\right)/CVC_{i}\;$$

In formula (1), LC_ij_ is the load coefficient of the j-th principal component and the i-th index, λ is the initial eigenvalue of j-th. In formula (2), VC is the variance contribution rate, and CVC is the cumulative variance contribution rate.

#### 2.2.2 Acquisition of hyperspectral data

**Figure 1:**
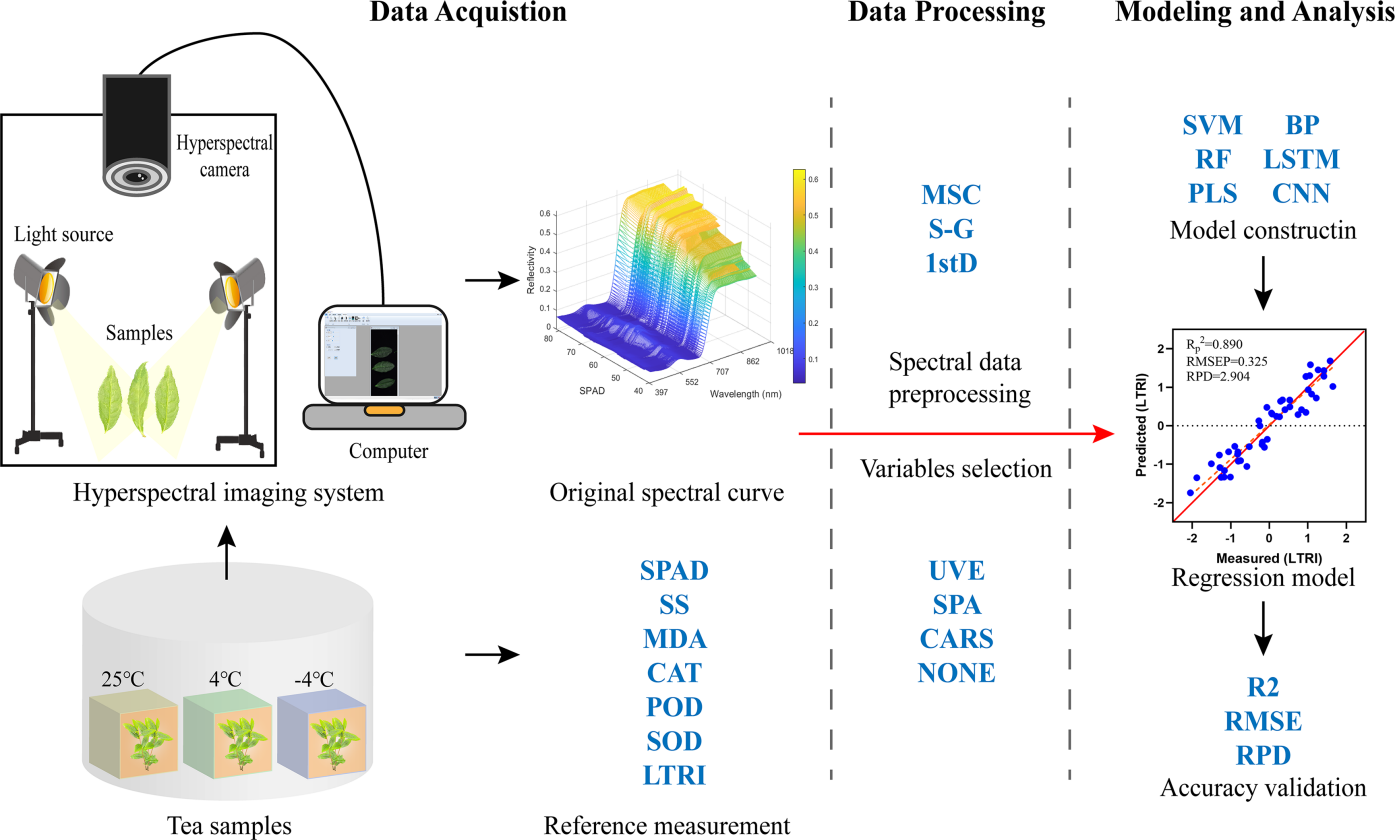
Acquisition and analysis of hyperspectral data. LTRI, low temperature response index; MSC, multivariate scattering correction; S-G, Savitzky-Golay; 1stD, first derivative.


The acquisition and analysis process of hyperspectral data was shown in **Figure 1**. In this study, HSI system (GaiaField-Pro, Jiangsu Dualix Spectral Image Technology Co. Ltd) was used to collect spectral information of tea leaves in the range of 397-1008 nm. The composition, specifications and parameters of the HSI system were the same as those in reference (Mao et al., 2022). The spatial resolution of hyperspectral images collected in this study was 960 × 1101 (Space × Spectrum), the spectral resolution was 3.5 nm and the number of spectral channels was 176. In order to reduce the influence of dark current noise and external environment, the original hyperspectral image (R) was corrected to reflectance hyperspectral image (C) by equation (3) after spectral data acquisition.

(3)  C=(R−D)/(W−D)

Where, C is the corrected image, R is the original image, D is the black reference image obtained by covering with a lens cover with a reflectance of about 0%, and W is the white reference image collected using a pure white standard whiteboard with a reflectance of about 100%.

### 2.3 Standardization and feature extraction of spectral data

The reflectance of hyperspectral image after black-and-white correction was opened in the SpecVIEW (Jiangsu Dualix Spectral Imaging, China) software, and the image was further corrected using tools such as lens and reflectance calibration to obtain a standardized hyperspectral image.

The background information of hyperspectral images was extracted by intensity threshold segmentation in the ENVI 5.3 (Research System Inc, America) software. Then, the entire tea leaf area in the hyperspectral image was defined as the region of interest (ROI), and the ROIs of all samples were extracted. Finally, the average reflectance of each sample ROI was calculated, resulting in a spectral matrix of 176 × 192 (variables × samples).

### 2.4 Data analysis methods

#### 2.4.1 Spectral preprocessing

Due to the influence of hyperspectral equipment and environmental factors, the spectrum of tea leaves has problems such as scattering effect and noise, which will weaken the spectral signals of the internal physical and chemical indicators of tea materials and affect the accuracy of the regression model. Therefore, in order to eliminate the spectral differences caused by different scattering levels, the MSC (Cheng et al., 2014) algorithm was used to remove artifacts or defects in the data matrix; In order to effectively reduce the random noise of the average reflection spectrum, the S-G (Kong et al., 2014) algorithm was used to “average” or “fit” each point within a certain width window, so as to obtain the best estimated value of the smooth point; In order to eliminate the process of baseline shift and split overlapping spectral peaks, the spectral band characteristics were enhanced by the 1^st^D (Feng and Sun, 2013). Among them, the formula of differential method-1^st^D is shown in formula (4).


(4) $$d_{y}/d_{x}=\left(y_{i+1}-y_{i}\right)/\Delta x\;$$

Where, y_i_ is the spectrum of the i-th sample, and Δx is the wavelength interval.

#### 2.4.2 Extraction of feature bands

Although the preprocessed spectral data has removed some noise, it still contains too much band information. This not only increased the amount of data operations, but more importantly, the redundancy of the band variables would affect the prediction accuracy and stability of the model (Ouyang et al., 2016). Therefore, SPA, CARS and UVE algorithms were used to select representative bands in the full-band spectral data as the “feature bands”, and compare them with the full-band (NONE) data, and finally select the best feature variable selection algorithm. Among them, the SPA algorithm can find the variable combination containing the least redundant information from the spectral information, and select a set of representative spectral variables with the minimum collinearity, thus reducing the complexity of the model (Araújo et al., 2001). The CARS algorithm is a variable screening method based on the principle of “survival of the fittest” in Darwin’s evolution theory, which can effectively find the optimal spectral combination (Yuan et al., 2021). The UVE algorithm can remove irrelevant variables with more noise, optimize model variables, and improve the predictive ability of the model (Li et al., 2016b). The basic parameters of SPA, CARS and UVE algorithms used in this study were shown in the **Table 2**.

**Table 2:** The parameters of feature band selection algorithms.

Algorithm	Parameters	Value
SPA	Minimum	1
	Maximum	30
	Epochs	20
CARS	Method	None
	Fold	10
	Number of PCA	10
	Monte Carlo sampling times	300
UVE	Optimal factor number	5
	Leave-One-Out	700
	Cutoff	0.99

#### 2.4.3 Model establishment

In order to find an optimal prediction model, a regression model between the spectral data of tea leaves and its physical and chemical indexes was established by using four machine learning methods of PLS, SVM, RF, BP and two deep learning methods of CNN and LSTM, and the effects of six models were compared.

Each of these six methods has its own advantages. Among them, the PLS algorithm is the widely used prediction model at present, it can not only overcome the collinearity problem, but also remove the influence of unhelpful noise on regression (Geladi and Kowalski, 1986). The optimal number of potential components in PLS was determined by minimizing the RMSE of leave-one-out cross-validation (Liu et al., 2021). The SVM can solve the classification and regression problems of high-dimensional features (Cortes and Vapnik, 1995). When applying a SVM to the regression problem, the model can be optimised by adjusting the polynomial kernel of a kernel function. The RF algorithm adopts random sampling, and the trained model has small variance and strong generalization ability (Breiman, 2001). The BP neural network has a high degree of self-learning and self-adaptive ability, and has the ability to apply learning results to new knowledge (Sun et al., 2019). The CNN network is one of the most effective networks for data feature extraction (Li et al., 2016a). The LSTM network can not only discover the mutual dependence of the data in the time series data, but also automatically detect the best mode for the relevant data (Hochreiter and Schmidhuber, 1997).

The network structures of PLS, SVM, RF, BP, CNN and LSTM were determined with reference to our two recent studies (Li et al., 2022a; Li et al., 2022b). Because the network structure applied in previous studies have made better progress in the monitoring agronomic traits of tea plants, and the established models have a strong generalization ability and are applicable to tea plants. Specifically, the input layer of the CNN is a two-dimensional matrix of spectral data. The hidden layer mainly consists of three convolutional layers, three activation functions, one maximum pooling layer, and one fully connected layer. In addition, the optimal combination of parameters was determined by continuously adjusting the network parameters. The final determined specific parameters were shown in **Table 3**.

**Table 3:** Main parameters of the PLS, SVM, RF, BP, CNN, and LSTM models.

Model	Model parameters	Value
PLS	N_components (Number of Components to Keep)	2
	Max iter (The Maximum Number of Iterations)	500
	Tol (Tolerance Used in the Iterative Algorithm)	10^-6^
	Scale (Scale the Data)	True
SVM	The Kernel Function	Polynomial Kernel
	Cache_size	200
	Tol (Tolerance Used in the Iterative Algorithm)	10^-3^
	Max_iter	-1
	C (Regularization Parameter)	1
RF	n_estimators (Number of Trees in the Forest)	200
	n_jobs	1
	Min_samples_leaf	5
	Min_impurity_split	0
	FBoot	1
BP	Batch size	64
	Epochs	400
	Learning Rate	0.01
	Goal	10^-5^
	Training Function	Gradient Descent
CNN	Normalize	L2
	Optimizer	Adam (Adaptive moment estimation)
	The Activation Function	ReLU (Rectified Linear Unit)
	Batch Size	64
	Learning Rate	0.001
	Epochs	400
	Dropout	0.5
	Verbose	1
LSTM	Normalize	L2
	Optimizer	Adam (Adaptive moment estimation)
	The Activation Function	Tanh (TanHyperbolic)
	NumHiddenUnits	20
	Batch Size	64
	Learning Rate	0.001
	Epochs	40
	Dropout	0.5
	Verbose	1

### 2.5 Test environment and model evaluation data analysis methods

Conditions for processing data in this experiment are as follows. Hardware, processor: Inter(R) Core (TM) i7-6700HQ CPU GHz 2.60 GHz (2 processors); machine RAM: 8GB. Software environment, MATLAB 2020; IBM SPSS Statistics 25.0; operating system: Windows 7.

To verify the accuracy of the algorithm, five-fold cross-validation was used in this study. The data set was divided into five parts, 4 parts were used for training sets and 1 part was used for testing set. That is, the training set included of 153 samples, and the testing set consisted of 39 samples. The data set was repeated 4 times, and then the results were averaged. In order to evaluate the performance of the model, the determination coefficient (R^2^) of calibration ( ${\text{R}}_{\text{c}}^{2}$ ) and prediction ( ${\text{R}}_{\text{p}}^{2}$ ), root mean square error (RMSE) of calibration (RMSEC), validation (RMSEV) and prediction (RMSEP), and relative predictive deviation (RPD) were used. Higher values of R^2^ generally indicate higher levels of model accuracy. The larger the RPD, the better performance of the model. On the contrary, lower values of RMSE indicate higher levels of model accuracy. R^2^, RMSE and RPD are calculated as follows: \


(5) $$R_{c}^{2},R_{p}^{2}=1-\sum_{i=1}^{n}{\left({\hat{y}}_{i}-y_{i}\right)}^{2}/\sum_{i=1}^{n}{\left(y_{i}-\bar{y}\right)}^{2}$$

(6) $$RMSEC,RMSEV,RMSEP=\sqrt{\sum_{i=1}^{n}{\left({\hat{y}}_{i}-y_{i}\right)}^{2}/n\;}$$

(7) $$\;\;\;\;\;\;\;\;\;\;\;RPD\;=1/\sqrt{1-R_{p}^{2}}$$

Where, n is the number of samples in the corresponding data set (calibration, verification and prediction), ${\hat{y}}_{i}$ and y_i_ are the predicted and measured values of the i-th tea samples, respectively, and $\bar{y}$ is the average measured value of the sample.

## 3 Results and discussion

### 3.1 Response of tea plants to low temperature stress

The statistical analysis of the measured physiological indexes of all tea samples (**Table 4**), including the maximum value, minimum value, average value and standard deviation.

**Table 4:** Descriptive statistics of low temperature-induced components and degree of low temperature damage in tea leaves.

Index	Maximum	Minimum	Average value	Standard deviation
SPAD	81.267	39.675	62.187	7.837
SS (mg/g)	39.120	17.000	28.524	5.323
MDA (nmol/g)	39.703	16.000	27.238	5.779
CAT (μmol/min/g)	38.170	17.159	26.469	4.884
POD (U/g)	12.700	1.000	5.973	2.908
SOD (U/g)	438.268	242.000	359.429	36.312

**Figure 2:**
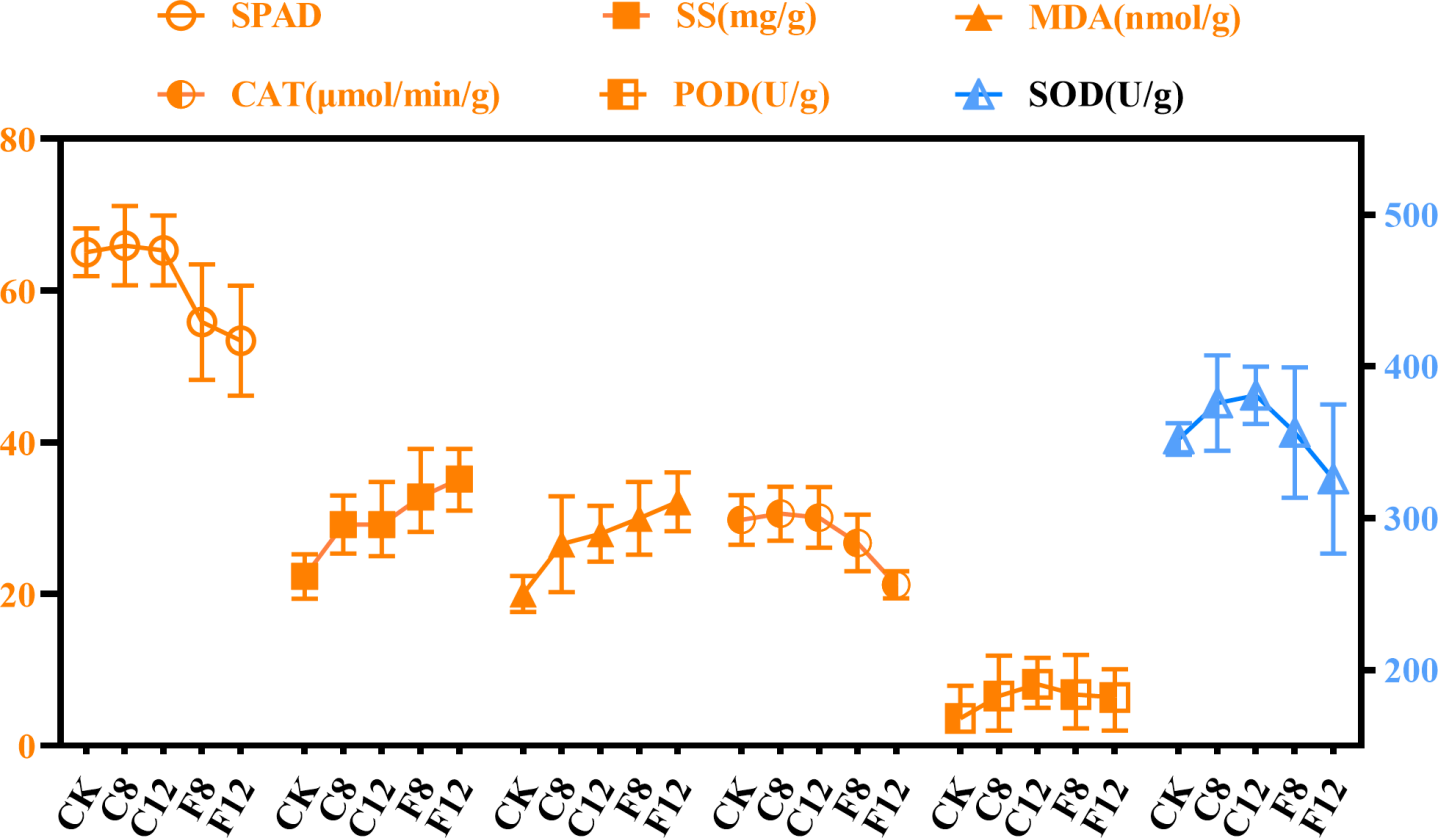
Data changes of low temperature-induced components under different low temperature conditions.

In this study, the change rules of 6 indicators were analyzed (**Figure 2**). The results showed that with the extension of low temperature time and the decrease of temperature, the SPAD value showed a general trend of decreasing. The contents of SS and MDA showed an upward trend. The activities of CAT, POD and SOD increased first and then decreased.

In general, under -4°C treatment, the SPAD value decreased more greatly, which may be due to the large degradation of chlorophyll after decreasing from 4°C to -4°C (Lajolo and Marquez, 1982). SS was an important substance in plant osmoregulation. The change of SS in this paper was consistent with the results of Zeng et al (Zeng et al., 2017). This may be because under low temperature stress, plant cells can resist low temperature by accumulating SS, increasing the concentration of cytosol and protecting the cytoplasm from freezing (Morgan, 1984). MDA was the product of membrane lipid peroxidation of plant organs under low temperature stress, which destroyed the stability of biofilms and could reflect the degree of damage to plant cell membranes (Liu et al., 2012). Therefore, the lower the temperature, the higher the MDA content, that was, the lower the temperature, the more serious the damage to the tea plants cell membrane. CAT, POD and SOD were significant protective enzymes in the plant membrane lipid peroxidase defense system. In this study, the changes in the activities of the three protective enzymes were basically the same, which was consistent with the previous research results of researchers on tea leaves (Li et al., 2014; Li et al., 2020).

### 3.2 Establishment of LTRI

The contents of SPAD (X_1_), SS (X_2_) and MDA (X_3_) and the activities of enzymes CAT (X_4_), POD (X_5_) and SOD (X_6_) can be used as effective indexes to reflect the low temperature stress of tea plants. Standardized processing and principal component analysis were performed on these 6 indicators using SPSS 25.0 software, and the total variance explanation of the principal components (**Table 5**) and each factor loading (**Table 6**) were obtained. The results showed that among the 6 `components, only the first two principal components satisfied the principle that the eigenvalue root (λ) was greater than 1, and can explain 85.231% of the data, which could reflect most of the information of the 6 low temperature-induced components. Therefore, the first two principal components (Y_1_ and Y_2_) can be applied to comprehensively analyze the low temperature stress of tea plants, which not only reduces the number of indicators, but also re-establishes the internal relationship among all low temperature-induced indicators (Han et al., 2020).

**Table 5:** The eigenvalues and variance contribution rate of each index correlation matrix.

Component(Y)	λ	VC(%)	CVC(%)
1	2.862	47.706	47.706
2	2.251	37.524	85.231
3	0.595	9.915	95.146
4	0.198	3.295	98.44
5	0.081	1.351	99.791
6	0.013	0.209	100

**Table 6:** Factor loadings in principal components.

	Y_1_	Y_2_
X_1_	0.826	-0.482
X_2_	-0.106	0.927
X_3_	0.638	0.732
X_4_	-0.562	-0.724
X_5_	-0.818	0.231
X_6_	0.881	-0.215

According to the data in **Table 5** and **Table 6**, the LCC of each parameter variable was calculated by formula (1) to obtain the linear composite expression of the first two principal components:


$$Y_{1}=0.488X_{1}-0.063X_{2}+0.377X_{3}-0.322X_{4}-0.484X_{5}+0.521X_{6}$$

$$\;Y_{2}=-0.321X_{1}+0.618X_{2}+0.488X_{3}-0.483X_{4}+0.154X_{5}-0.143X_{6}$$

CSC was calculated by formula (2) to obtain the comprehensive model of principal component (Y):


$$\;\;\;\;\;\;Y=0.132X_{1}+0.237X_{2}+0.426X_{3}-0.398X_{4}-0.203X_{5}+0.228X_{6}$$

Based on the percentage method, each coefficient of the above comprehensive scoring model was normalized to obtain LTRI:


$$LTRI=0.081X_{1}+0.146X_{2}+0.262X_{3}-0.245X_{4}-0.125X_{5}+0.141X_{6}$$

**Figure 3:**
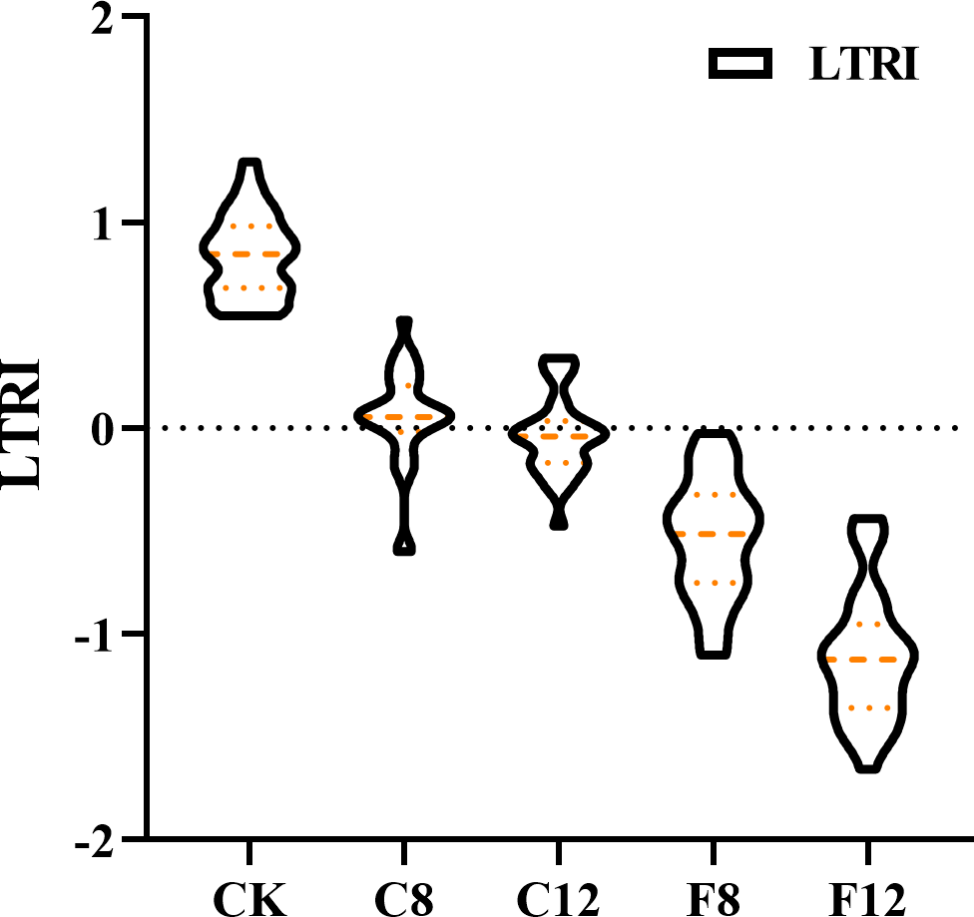
Variation of low temperature response index (LTRI) under different low temperatures.

The hypothermia response map of LTRI (**Figure 3**) showed that the change of LTRI was closely related to the duration of hypothermia and the severity of freezing injury. The smaller the LTRI value, the greater the damage of low temperature to tea plants. It was speculated that LTRI could be used to comprehensively evaluate the cold resistance response of tea plants.

### 3.3 Preprocessing of spectral data

**Figure 4:**
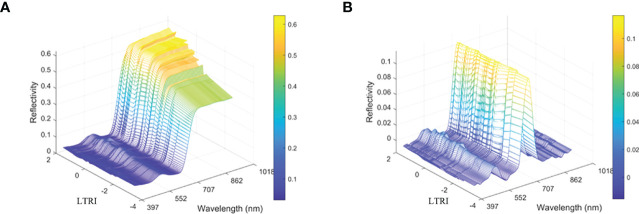
Image comparison of unprocessed spectral data and preprocessed spectral data. **(A)** Represent the original spectrum **(B)** Represent pretreatment spectrum.

In addition to the information related to the samples, the raw spectral data also contains baseline drift, noise and other information, which reduces the robustness and accuracy of prediction or classification models (Pu et al., 2014). In order to establish a stable and reliable quantitative analysis model, S-G, MSC and 1^st^D were combined to preprocess the spectral data. The original average reflectance spectrum and the spectral curve after preprocessing were shown in **Figure 4**. After pretreatment, the absorption peaks and reflection valleys of the spectrum can be clearly observed. The absorbance of tea samples increased at 650 nm and 800 nm.

### 3.4 Selection and analysis of characteristic bands

The spectral data obtained includes 176 bands. Considering the redundancy between variables, CARS, SPA and UVE algorithms were used to select feature bands to reduce useless information, thereby improving the efficiency and reliability of the model. The number and distribution of characteristic bands were shown in **Table 7** and **Supplementary Table 1**.

**Table 7:** Bands screening result.

	Screening method			
	CARS	SPA	UVE	NONE
Index	Number of bands			
SPAD	11	16	82	176
SS (mg/g)	13	16	112	176
MDA (nmol/g)	19	10	81	176
CAT (μmol/min/g)	6	11	70	176
POD (U/g)	19	13	83	176
SOD (U/g)	11	17	15	176
LTRI	17	16	150	176

The results showed that among the characteristic band screening methods of SPAD, UVE had the largest number of feature bands, which was 82, and CARS had the least number of feature bands, which was 11. Among the feature band screening methods of SS, UVE had the largest number of feature bands, which was 112, and CARS had the least number of feature bands, which was 13. Among the characteristic band screening methods of MDA, UVE had the largest number of feature bands, which was 81. The number of characteristic bands screened by SPA was the least, which was 10; Among the feature band screening methods of CAT, UVE had the largest number of characteristic bands, 70, and the number of characteristic bands filtered by CARS was the least, 6; Among the characteristic band screening methods of POD, UVE had the largest number of characteristic bands, 83, and SPA had the least number of characteristic bands, 13; Among SOD’s characteristic band screening method, SPA had the largest number of characteristic bands, 17, and CARS had the largest number of characteristic bands, 11; Among the characteristic band screening methods of LTRI, UVE had the largest number of feature bands, 150, and SPA had the least number of feature bands, 16.

Overall, CARS and SPA haven better variable selection ability than UVE, the UVE algorithm filters more abundant feature bands than CARS and SPA. The study by Ji et al (Ji et al., 2022). also showed that compared with other feature band selection methods, the number of features obtained by UVE selection was the largest.

### 3.5 Establishment and comparison of models

#### 3.5.1 Analysis of the best models for different indicators

**Figure 5:**
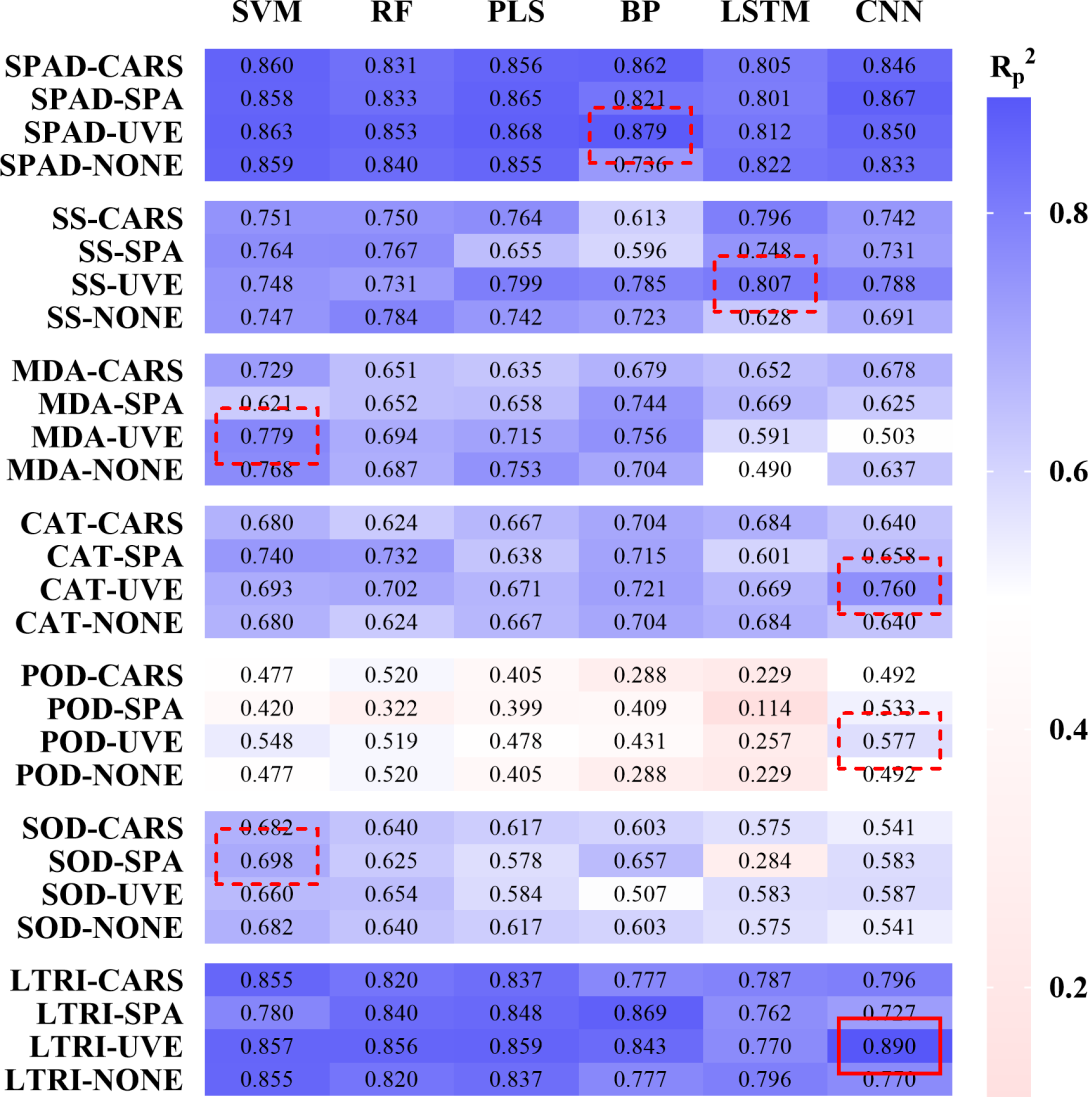
Modeling results of low temperature-induced components and low temperature injury degree of tea leaves.

**Figure 6:**
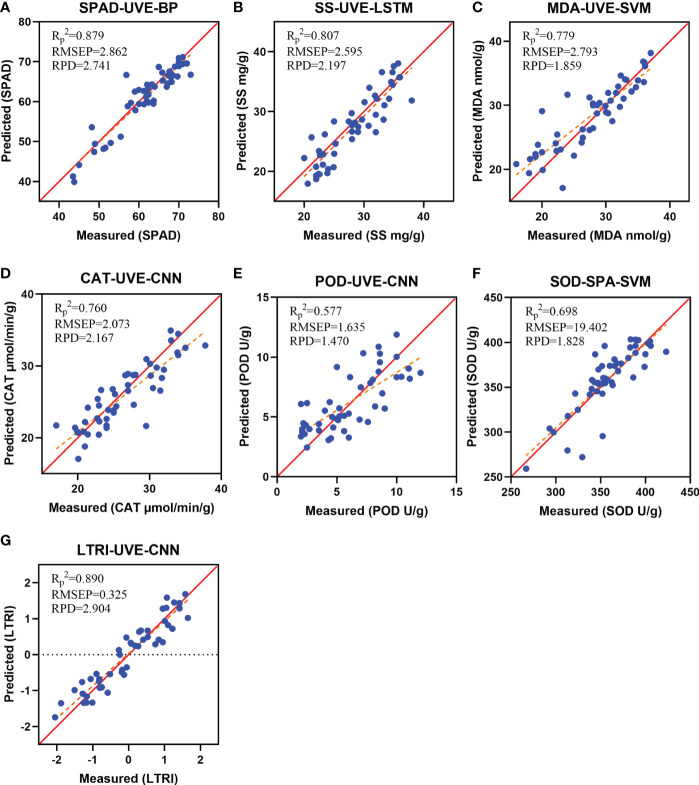
Scatter plot of measured and predicted values. **(A)** SPAD-UVE-BP; **(B)** SS-UVE-LSTM; **(C)** MDA-UVE-SVM; **(D)** CAT-UVE-CNN; **(E)** POD-UVE-CNN; **(F)** SOD-SPA-SVM; **(G)** LTRI-UVE-CNN.

Based on the characteristic bands screened by CARS, SPA, UVE algorithms and all the bands, a total of 168 different indexes models were established by using PLS, SVM, RF, BP, LSTM and CNN algorithms. The accuracy evaluations (R^2^, RMSE and RPD) of all models were shown in **Supplementary Table 2**. **Figure 5** showed the validation results of the model with ${\text{R}}_{\text{p}}^{2}$ evaluation test set samples. The results indicated that in the prediction of SPAD, SS, MDA, CAT, POD, SOD and LTRI, the models with the highest prediction accuracy were SPAD-UVE-BP, SS-UVE-LSTM, MDA-UVE-SVM, CAT-UVE- CNN, POD-UVE-CNN, SOD-SPA-SVM and LTRI-UVE-CNN, the ${\text{R}}_{\text{p}}^{2}$ of the models were 0.879, 0.807, 0.779, 0.760, 0.577, 0.698 and 0.890, respectively. **Figure 6** showed the prediction results of the best model for 7 indicators. Among them, the red line was the 1:1 line, and the orange line was the regression line between the predicted and actual values. The predicted values of the samples have been distributed around the regression line at a relatively close distance, indicating that the 7 models have good robustness.

To sum up, the LTRI comprehensive model had higher accuracy and better efficacy than the 6 single index models. This demonstrated that the relationship between LTRI and spectrum was closer than other single physicochemical indicators. LTRI can more comprehensively and objectively evaluate the low temperature stress of tea plants, and effectively evaluate the cold resistance of tea plants.

#### 3.5.2 Comparison of different variable selection methods based on LTRI

**Figure 7:**
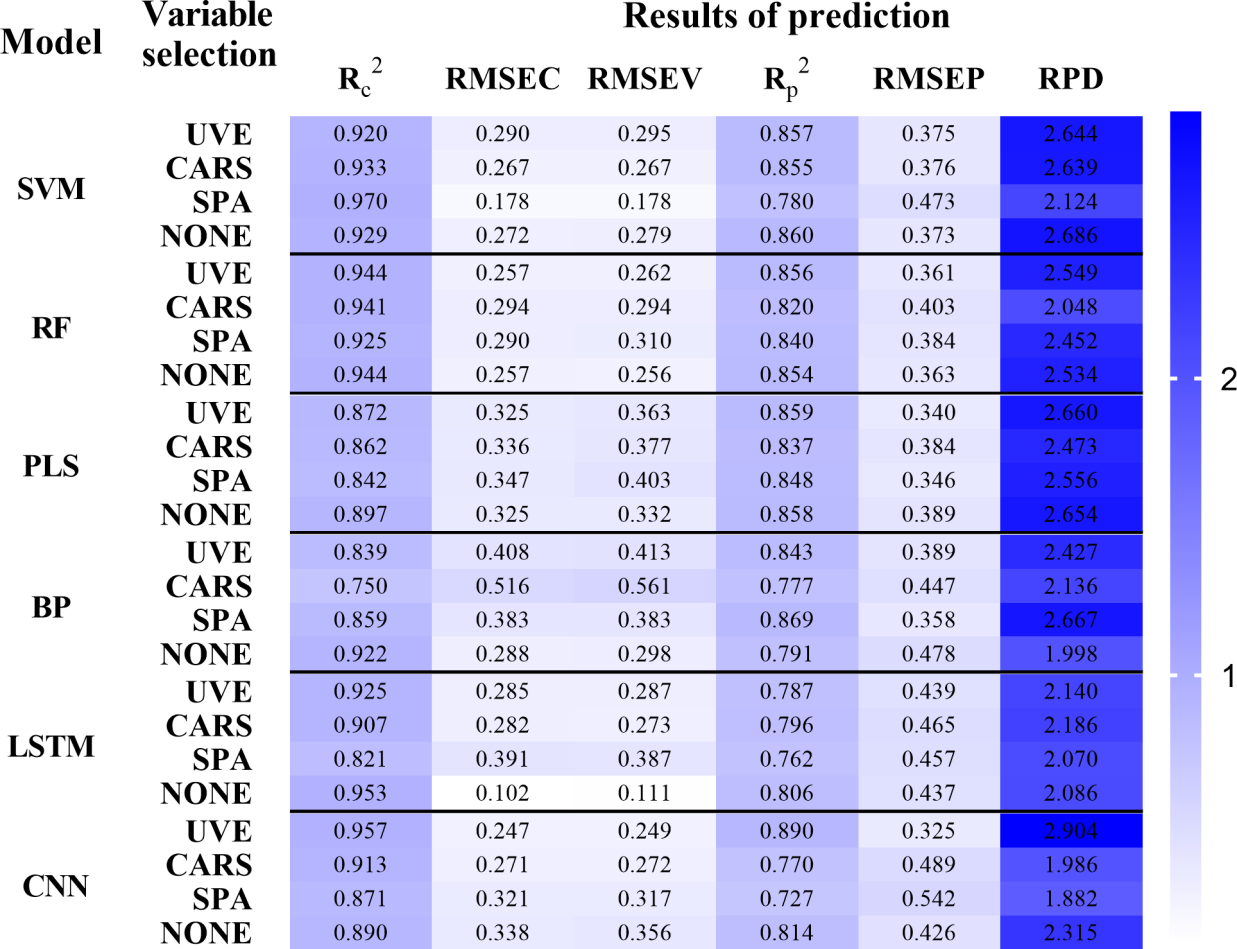
Prediction results of the tea plant low temperature response index (LTRI) model based on the characteristic bands selected by different variable selection methods.

Since LTRI was more representative in each indicator, this section only described the prediction model based on LTRI. **Figure 7** showed the comparison of the results of the LTRI models established based on the different number of characters bands. The results showed that the spectral information of the bands selected by the UVE algorithm was more abundant and effective, and the regression effect of the model based on the characteristic bands screened by the UVE was better than the model based on the full-band and the models based on the feature bands screened by CARS and SPA. The study of Yang et al (Yang et al., 2021). also proved that the characteristic wavelength model established after variable screening was better than the full-band model. The reasons may be: compared with the full-band, UVE deletes some collinearity variables; compared with CARS and SPA algorithms, UVE algorithm retains more information related to LTRI in spectral data. In addition, the CARS may cause unstable wavelength selection results due to Monte Carlo sampling (Yun et al., 2014). While the SPA greatly reduces the number of variables and simplifies the model, it may also delete some key variables, thus reducing the accuracy of predictions. The research results of Ji et al (Ji et al., 2022). also showed that CARS extracted fewer feature bands than UVE-SPA, but also lost more useful bands, resulting in a worse subsequent regression effect. This was consistent with the findings of Guo et al (Guo et al., 2020). They compared the results of the UVE-SPA-PLS and CARS-SPA-PLS models with those of the UVE-PLS and CARS-PLS models, and found that the predictive performance of the SPA engagement model was slightly lower than that of UVE-PLS and CARS-PLS.

#### 3.5.3 Comparison of different modeling methods based on LTRI

In order to compare the prediction effects of different modeling algorithms on LTRI, 18 prediction models of LTRI were analysed and compared. As shown in **Figure 7**, among the 6 modeling algorithms, the CNN model had the best performance (UVE-CNN, ${\text{R}}_{\text{p}}^{2}$ = 0.890). Interestingly, the other two combined models of CNN (CARS-CNN, ${\text{R}}_{\text{p}}^{2}$ = 0.770; SPA-CNN, ${\text{R}}_{\text{p}}^{2}$ = 0.727) achieved poor results. On the one hand, because these three CNN models all use different types of input vectors, and the feature bands obtained based on UVE screening were more than that of SPA and CARS. Thus, the UVE-CNN model included more characteristic information, which indirectly improved the accuracy of CNN model prediction (Hsieh and Kiang, 2020). On the other hand, because deep learning was very suitable for a large number of data sets, there were only 192 samples in this paper, which limited the use of deep learning to a certain extent. Even so, the ${\text{R}}_{\text{p}}^{2}$ of all models in this study exceeded 0.70, indicating that model performance can be improved in future studies. Perhaps, the expansion of the sample set can expand the advantages of the deep learning method. In addition, other deep learning models (Stacked Auto-Encoders, Recurrent Neural Network, and Deep Belief Networks) may be interesting explorations for the evaluation of tea plants frost damage.

In summary, the LTRI-UVE-CNN model achieves the best results ( ${\text{R}}_{\text{c}}^{2}$ = 0.957, RMSEC=0.247, RMSEP=0.249, ${\text{R}}_{\text{p}}^{2}$ = 0.890, RMSEP=0.325, RPD=2.904), which proves that the comprehensive evaluation model is better than the single physical and chemical index model, it is also proved that the UVE-CNN architecture can exhibit higher prediction performance.

## 4 Conclusion

In this study, machine learning and deep learning models for 7 low temperature stress evaluation indicators, including SPAD, SS, MDA, CAT, POD, SOD and LTRI were established based on the hyperspectral data of tea leaves under low temperature stress, through spectral preprocessing, feature-band selection and other methods.

The results showed that it is possible to rapidly, non-destructively and accurately assess the freezing injury of tea plants by combining appropriate variable selection methods and modelling algorithms. The best prediction models for the 7 indicators were SPAD-UVE-BP, SS-UVE-LSTM, MDA-UVE-SVM, CAT-UVE-CNN, POD-UVE-CNN, SOD-SPA-SVM and LTRI-UVE-CNN, ${\text{R}}_{\text{p}}^{2}$ was 0879, 0.807, 0.779, 0.760, 0.577, 0.698 and 0.890, respectively. Among them, the LTRI model was superior to the single physical and chemical index evaluation model, and more suitable for the comprehensive evaluation of low temperature stress to tea plants. In addition, compared with the three variable selection methods (i.e., CARS, UNE and SPA), the UVE method performed best; compared with 6 modeling algorithms (i.e., PLS, SVM, RF, BP, CNN and LSTM), the CNN algorithm performed best.

In conclusion, the LTRI values constructed by principal component analysis in this study can effectively evaluate tea plants response to the low temperature stress. The CNN model of LTRI based on the selected bands of UVE was proved to be accurate and robust (LTRI-UVE-CNN: ${\text{R}}_{\text{p}}^{2}$ = 0.890, RMSEP = 0.325, RPD = 2.904). This study provides the basis for non-destructive and accurate monitoring of tea plants under natural disaster by using hyperspectral imaging and modeling algorithms.

## Data availability statement

The original contributions presented in the study are included in the article/**Supplementary Material**. Further inquiries can be directed to the corresponding authors.

## Author contributions

YM carried out the experiment, collected and organized data, provided the article’s picture, and wrote the manuscript. HL use multiple models to analyze the data. LS and JS embellish the language of this article. HL, LS, JS, JZ, XH, YS and CB participated in the design of the experiment and directed the study. ZD, YW, and KF proposed the hypothesis for this work, designed the experiment, helped organize the manuscript structure and directed the study. All authors contributed to the article and approved the submitted version.

## Supplementary material

Supplementary Table 1 Bands screening results.

Supplementary Table 2 Modeling results.

## References

[B1] Araújo M. Saldanha T. Galvao R. Yoneyama T. Chame H. Visani V. (2001). The successive projections algorithm for variable selection in spectroscopic multicomponent analysis. Chemometr. intell. Lab. Syst. 57 (2), 65–73. doi: 10.1016/S0169-7439(01)00119-8

[B2] Ban Q. Wang X. Pan C. Wang Y. Kong L. Jiang H.. (2017). Comparative analysis of the response and gene regulation in cold resistant and susceptible tea plants. PloS One 12 (12), e0188514. doi: 10.1371/journal.pone.0188514 29211766 PMC5718485

[B3] Breiman L. (2001). Random forests. Mach. Learn. 45, 5–32. doi: 10.1023/A:1010933404324

[B4] Chen D. Zhang F. Tan M. Chan N. Shi J. Liu C.. (2022). Improved na+ estimation from hyperspectral data of saline vegetation by machine learning. Comput. Electron. Agric. 196, 106862. doi: 10.1016/j.compag.2022.106862

[B5] Chen S. Gao Y. Fan K. Shi Y. Luo D. Shen J.. (2021). Prediction of drought-induced components and evaluation of drought damage of tea plants based on hyperspectral imaging. Front. Plant Sci. 12. doi: 10.3389/fpls.2021.695102 PMC8417055 34490000

[B6] Cheng J. Sun D. Zeng X. Pu H. (2014). Non-destructive and rapid determination of TVB-n content for freshness evaluation of grass carp (Ctenopharyngodon idella) by hyperspectral imaging. Innov. Food Sci. Emerg. Technol. 21, 179–187. doi: 10.1016/j.ifset.2013.10.013

[B7] Cortes C. Vapnik V. (1995). Support-vector networks. Mach. Learn. 20 (3), 273–297. doi: 10.1007/BF00994018

[B8] Duan D. Zhao C. Li Z. Yang G. Yang W. (2019). Estimating total leaf nitrogen concentration in winter wheat by canopy hyperspectral data and nitrogen vertical distribution. J. Integr. Agric. 18 (7), 1562–1570. doi: 10.1016/S2095-3119(19)62686-9

[B9] Feng M. Guo X. Wang C. Yang W. Shi C. Ding G.. (2018). Monitoring and evaluation in freeze stress of winter wheat (Triticum aestivum l.) through canopy hyperspectrum reflectance and multiple statistical analysis. Ecol. Indic. 84, 290–297. doi: 10.1016/j.ecolind.2017.08.059

[B10] Feng Y. Sun D. (2013). Near-infrared hyperspectral imaging in tandem with partial least squares regression and genetic algorithm for non-destructive determination and visualization of pseudomonas loads in chicken fillets. Talanta 109, 74–83. doi: 10.1016/j.talanta.2013.01.057 23618142

[B11] Geladi P. Kowalski B. (1986). Partial least-squares regression: A tutorial. Analytica chimica Acta 185, 1–17. doi: 10.1016/0003-2670(86)80028-9

[B12] Guo Z. Wang M. Shujat A. Wu J. El-Seedi H. Shi J.. (2020). Nondestructive monitoring storage quality of apples at different temperatures by near-infrared transmittance spectroscopy. Food Sci. Nutr. 8 (7), 3793–3805. doi: 10.1002/fsn3.1669 32724641 PMC7382128

[B13] Han B. Ma X. Cui D. Wang Y. Geng L. Cao G. (2020). Comprehensive evaluation and analysis of the mechanism of cold tolerance based on the transcriptome of weedy rice seedlings. Rice 13 (1), 1–14. doi: 10.1186/s12284-019-0363-1 32056019 PMC7018935

[B14] Hochreiter S. Schmidhuber J. (1997). Long short-term memory. Neural Comput. 9 (8), 1735–1780. doi: 10.1162/neco.1997.9.8.1735 9377276

[B15] Hsieh T. Kiang J. (2020). Comparison of CNN algorithms on hyperspectral image classification in agricultural lands. Sensors 20 (6), 1734. doi: 10.3390/s20061734 32244929 PMC7146316

[B16] Ji J. Li P. Jin X. Ma H. Li M. (2022). Study on quantitative detection of tomato seeding robustness in spring seeding transplanting period based on VIS-NIR spectroscopy. Spectr. Spectral Anal. 42 (06), 1741–1748. doi: 10.3964/j.issn.1000-0593(2022)06-1741-08

[B17] Kong W. Liu F. Zhang C. Bao Y. Yu J. He Y. (2014). Fast detection of peroxidase (POD) activity in tomato leaves which infected with botrytis cinerea using hyperspectral imaging. Spectrochimica Acta Part A: Mol. Biomol. Spectrosc. 118, 498–502. doi: 10.1016/j.saa.2013.09.009 24080581

[B18] Lajolo F. Marquez U. L. (1982). Chlorophyll degradation in a spinach system at low and intermediate water activities. J. Food Sci. 47 (6), 1995–1998. doi: 10.1111/j.1365-2621.1982.tb12929.x

[B19] Li H. Mao Y. Wang Y. Fan K. Shi H. Sun L.. (2022a). Environmental simulation model for rapid prediction of tea seedling growth. Agronomy 12 (12), 3165. doi: 10.3390/agronomy12123165

[B20] Li H. Wang Y. Fan K. Mao Y. Shen Y. Ding Z. (2022b). Evaluation of important phenotypic parameters of tea plantations using multi-source remote sensing data. Front. Plant Sci. 13, 898962. doi: 10.3389/fpls.2022.898962 35937382 PMC9355610

[B21] Li S. Xu W. Hui X. Gu B. Jie C. (2011). Quantifying carbon storage for tea plantations in China. Agric. Ecosyst. Environ. 141 (3), 390–398. doi: 10.1016/j.agee.2011.04.003

[B22] Li Y. Hao Z. Lei H. (2016a). Survey of convolutional neural network. J. Comput. Appl. 36 (9), 2508. doi: 10.11772/j.issn.1001-9081.2016.09.2508

[B23] Li Y. Ma B. Li C. Yu G. (2022c). Accurate prediction of soluble solid content in dried hami jujube using SWIR hyperspectral imaging with comparative analysis of models. Comput. Electron. Agric. 193, 106655. doi: 10.1016/j.compag.2021.106655

[B24] Li Y. Shu X. Zhou Y. Jiang C. (2014). Change in physiological characteristics and cold resistance evaluation of three cultivars of camellia sinensis during natural overwintering period. Plant Resour. Environ. 23 (3), 7. doi: 10.16590/j.cnki.1001-4705.2020.12.038

[B25] Li Y. Tian W. Xie E. Cen L. Zhao D. (2020). Comprehensive evaluation of cold-resistant camellia sinensis (L.)O. ktze. based on physiological and biochemical indexes under low temperature stress. Seed 12, 38–43+54.

[B26] Li Z. Wang J. Xiong Y. Li Z. Feng S. (2016b). The determination of the fatty acid content of sea buckthorn seed oil using near infrared spectroscopy and variable selection methods for multivariate calibration. Vibrational Spectrosc. 84, 24–29. doi: 10.1016/j.vibspec.2016.02.008

[B27] Liu S. Jin X. Nie C. Wang S. Yu X. Cheng M.. (2021). Estimating leaf area index using unmanned aerial vehicle data: shallow vs. deep machine learning algorithms. Plant Physiol. 187 (3), 1551–1576. doi: 10.1093/plphys/kiab322 34618054 PMC8566226

[B28] Liu W. Zheng C. Chen J. Qiu J. Huang Z. Wang Q.. (2019a). Cold acclimation improves photosynthesis by regulating the ascorbate–glutathione cycle in chloroplasts of kandelia obovata. J. Forestry Res. 30 (3), 755–765. doi: 10.1007/s11676-018-0791-6

[B29] Liu X. Sun J. Ding P. Zhang J. Guo W. Liu L. (2012). The effect of low temperature stress on endogenous hormones in phalaenopsis. Acta Agriculturae Universitatis Jiangxiensis 34 (3), 464–469.

[B30] Liu Y. Wang Q. Gao X. Xie A. (2019b). Total phenolic content prediction in flos lonicerae using hyperspectral imaging combined with wavelengths selection methods. J. Food Process Eng. 42 (6), e13224. doi: 10.1111/jfpe.13224

[B31] Mao Y. Li H. Wang Y. Fan K. Song Y. Han X.. (2022). Prediction of tea polyphenols, free amino acids and caffeine content in tea leaves during wilting and fermentation using hyperspectral imaging. Foods 11 (16), 2537. doi: 10.3390/foods11162537 36010536 PMC9407140

[B32] Morgan J. (1984). Osmoregulation and water stress in higher plants. Annu. Rev. Plant Physiol. 35, 299–319. doi: 10.1146/annurev.pp.35.060184.001503

[B33] Ouyang Q. Zhao J. Pan W. Chen Q. (2016). Real-time monitoring of process parameters in rice wine fermentation by a portable spectral analytical system combined with multivariate analysis. Food Chem. 190, 135–141. doi: 10.1016/j.foodchem.2015.05.074 26212952

[B34] Pan L. Lu R. Zhu Q. Tu K. Cen H. (2016). Predict compositions and mechanical properties of sugar beet using hyperspectral scattering. Food Bioprocess Technol. 9 (7), 1177–1186. doi: 10.1007/s11947-016-1710-5

[B35] Pu H. Sun D. Ma J. Liu D. Cheng J. (2014). Using wavelet textural features of visible and near infrared hyperspectral image to differentiate between fresh and frozen–thawed pork. Food Bioprocess Technol. 7 (11), 3088–3099. doi: 10.1007/s11947-014-1330-x

[B36] Qureshi A. Khan A. Zameer A. Usman A. (2017). Wind power prediction using deep neural network based meta regression and transfer learning. Appl. Soft Computing 58, 742–755. doi: 10.1016/j.asoc.2017.05.031

[B37] Sonobe R. Sano T. Horie H. (2018). Using spectral reflectance to estimate leaf chlorophyll content of tea with shading treatments. Biosyst. Eng. 175, 168–182. doi: 10.1016/j.biosystemseng.2018.09.018

[B38] Su Z. Zhang C. Yan T. Zhu J. Zeng Y. Lu X.. (2021). Application of hyperspectral imaging for maturity and soluble solids content determination of strawberry with deep learning approaches. Front. Plant Sci. 12. doi: 10.3389/fpls.2021.736334 PMC8462090 34567050

[B39] Sun Q. Zhang M. Yang P. (2019). Combination of LF-NMR and BP-ANN to monitor water states of typical fruits and during microwave vacuum drying. LWT 116, 108548. doi: 10.1016/j.lwt.2019.108548

[B40] Wang H. Huo Z. Zhou G. Liao Q. Feng H. Wu L. (2016). Estimating leaf SPAD values of freeze-damaged winter wheat using continuous wavelet analysis. Plant Physiol. Biochem. 98, 39–45. doi: 10.1016/j.plaphy.2015.10.032 26610092

[B41] Wang Y. Hu X. Hou Z. Ning J. Zhang Z. (2018). Discrimination of nitrogen fertilizer levels of tea plant (Camellia sinensis) based on hyperspectral imaging. J. Sci. Food Agric. 98 (12), 4659–4664. doi: 10.1002/jsfa.8996 29607500

[B42] Wang Y. Li Y. Wang J. Xiang Z. Xi P. Zhao D. (2021). Physiological changes and differential gene expression of tea plants (Camellia sinensis (L.) kuntze var. niaowangensis QH Chen) under cold stress. DNA Cell Biol. 40 (7), 906–920. doi: 10.1089/dna.2021.0147 34129383 PMC8309439

[B43] Weng S. Yu S. Guo B. Tang P. Liang D. (2020). Non-destructive detection of strawberry quality using multi-features of hyperspectral imaging and multivariate methods. Sensors 20 (11), 3074. doi: 10.3390/s20113074 32485900 PMC7308843

[B44] Yang C. Zhao Y. An T. Liu Z. Jiang Y. Li Y.. (2021). Quantitative prediction and visualization of key physical and chemical components in black tea fermentation using hyperspectral imaging. LWT 141, 110975. doi: 10.1016/j.lwt.2021.110975

[B45] Yuan R. Liu G. He J. Wan G. Fan N. Li Y.. (2021). Classification of lingwu long jujube internal bruise over time based on visible near-infrared hyperspectral imaging combined with partial least squares-discriminant analysis. Comput. Electron. Agric. 182, 106043. doi: 10.1016/j.compag.2021.106043

[B46] Yun Y. Wang W. Tan M. Liang Y. Li H. Cao D.. (2014). A strategy that iteratively retains informative variables for selecting optimal variable subset in multivariate calibration. Analytica chimica Acta 807, 36–43. doi: 10.1016/j.aca.2013.11.032 24356218

[B47] Zeng G. Zhou L. Li X. Wen Z. (2017). Changes in physiological and biochemical indexes and anatomical structure of leaf of camellia sinensis during natural overwintering period. J. Plant Environ. 26(1), 63–68

[B48] Zhang L. Zhang Q. Wu J. Liu Y. Yu L. Chen Y. (2022). Moisture detection of single corn seed based on hyperspectral imaging and deep learning. Infrared Phys. Technol. 125, 104279. doi: 10.1016/j.infrared.2022.104279

