# Supplementary material for: Low temperature response index for monitoring freezing injury of tea plant
Source: Front Plant Sci. 2023 Feb 2;14:1096490. doi: 10.3389/fpls.2023.1096490 (PMC9933980; doi:10.3389/fpls.2023.1096490)
Supplement: Supplementary Table 1 — Bands screening results. [file Table_1.docx]

| **Index** | **Screening method** | **Number of bands** | **Characteristic bands (nm)** |
| --- | --- | --- | --- |
| SPAD | UVE | 82 | 478-514, 628-658, 696-707, 717-724, 752-762, 780-791, 812-830, 869-884, 946-960, 982-1001 |
|  | CARS | 11 | 481, 693, 721-724, 734, 766, 791, 805, 869, 916, 938 |
|  | SPA | 16 | 397, 433, 488, 517, 554, 597, 652, 689, 734, 759, 769, 787, 898, 935, 949, 986 |
| SS | UVE | 112 | 397-517, 557-564, 693-700, 769-801, 823-848, 873-880, 891, 920-938, 986-997 |
|  | CARS | 13 | 442, 507, 527, 658, 801-805, 823, 837-840, 876, 916-920, 953 |
|  | SPA | 16 | 397, 433, 446, 488, 517, 557, 648, 700, 741, 755, 787, 812, 833, 851, 953, 1001 |
| MDA | UVE | 81 | 410-413, 423-429, 501-527, 560-604, 686-693, 766-777, 826-887, 938-942, 971, 1001 |
|  | CARS | 19 | 524, 567, 594-607, 655, 686, 714-717, 734, 794, 830, 873-876, 986-990, 1001 |
|  | SPA | 10 | 597, 672, 689, 734, 769, 833, 898, 935, 949, 994 |
| CAT | UVE | 70 | 498-531, 557-604, 682-689, 724-727, 858-891, 935, 964-975, 997-1001 |
|  | CARS | 6 | 478-481, 587, 628, 858, 1001 |
|  | SPA | 11 | 433, 449, 517, 672, 689, 898, 935, 949, 968, 990, 1001 |
| POD | UVE | 83 | 397-485, 689-693, 769-798, 924, 957-1001 |
|  | CARS | 19 | 468, 665, 780-784, 873-905, 938, 964, 1001 |
|  | SPA | 13 | 488, 517, 594, 676, 686, 731, 745, 769, 876, 905, 931, 953, 968 |
| SOD | UVE | 15 | 514-517, 679-689, 717-724, 766, 805-816 |
|  | CARS | 11 | 426, 471-475, 544-547, 597, 682, 707, 727, 801, 816 |
|  | SPA | 17 | 397, 429, 462, 517, 597, 653, 689, 734, 766, 791, 830, 844, 898, 931, 953, 968, 1001 |
| LTRI | UVE | 150 | 397-521, 584-594, 682-700, 752, 773, 819-844, 869-880, 924-931, 949-1001 |
|  | CARS | 17 | 449, 540-544, 591-594, 777, 837, 855-858, 876-880, 898-902, 935-938, 949, 1001 |
|  | SPA | 16 | 397, 433, 449, 488, 517, 597, 652, 689, 734, 759, 769, 787, 898, 935, 949, 896 |

Supplementary table 1: Distribution of characteristic bands.
