# Supplementary material for: Low temperature response index for monitoring freezing injury of tea plant
Source: Front Plant Sci. 2023 Feb 2;14:1096490. doi: 10.3389/fpls.2023.1096490 (PMC9933980; doi:10.3389/fpls.2023.1096490)
Supplement: Supplementary Table 2 — Modeling results. [file Table_2.pdf]

Index		UVE					
		**SVM**	**RF**	**PLS**	**BP**	**LSTM**	**CNN**
SPAD	RC2	0.9247	0.9614	0.9182	0.9138	0.9328	0.9174
	RMSEC	2.2437	1.6563	2.4930	2.5281	1.8402	2.4281
	RMSEV	2.2437	1.6563	2.3821	2.5281	1.8030	2.2810
	RP2	0.8626	0.8525	0.8678	0.8794	0.8124	0.8503
	RMSEP	2.9533	3.0362	2.9300	2.8616	4.1072	3.1042
	RPD	2.6817	2.5265	2.7471	2.7474	2.1855	2.9830
SS	RC2	0.7856	0.9074	0.7499	0.7433	0.8750	0.8196
	RMSEC	2.4413	1.7443	2.7200	2.6766	1.7147	2.0100
	RMSEV	2.4830	1.7831	2.7822	2.6861	1.8430	2.1100
	RP2	0.7483	0.7308	0.7985	0.7848	0.8070	0.7881
	RMSEP	2.6297	2.6024	2.2400	2.3533	2.5951	2.3800
	RPD	1.9490	1.6720	2.1217	2.1524	2.1947	2.1600
MDA	RC2	0.8916	0.8680	0.6741	0.6452	0.7320	0.6164
	RMSEC	1.8656	2.1843	3.2700	3.4024	3.1080	3.5108
	RMSEV	1.8656	2.1843	3.2242	3.4024	3.1120	3.5108
	RP2	0.7787	0.6941	0.7147	0.7562	0.5906	0.5027
	RMSEP	2.7932	3.1927	3.0500	2.9610	3.7779	3.9729
	RPD	1.8585	1.4235	1.6029	1.9601	1.3485	1.1850
CAT	RC2	0.7634	0.8864	0.6823	0.6907	0.8998	0.7737
	RMSEC	2.4618	1.7684	2.9300	2.8360	1.6960	2.1696
	RMSEV	2.4728	1.7814	2.8505	2.8360	1.7230	0.0696
	RP2	0.6925	0.7021	0.6710	0.7209	0.6688	0.7603
	RMSEP	2.8657	2.7409	2.8100	2.6081	2.9073	2.0730
	RPD	1.7368	1.6613	1.5374	1.8882	1.6734	2.1670
POD	RC2	0.6780	0.8697	0.5328	0.5562	0.5630	0.6770
	RMSEC	1.7014	1.1856	2.1300	1.9913	1.8360	1.7036
	RMSEV	1.7124	1.1856	2.0413	1.9913	1.8620	0.0036
	RP2	0.5480	0.5194	0.4776	0.4306	0.2569	0.5774
	RMSEP	1.9962	1.9850	2.2500	2.1730	2.7199	1.6350
	RPD	1.3500	1.0933	1.1306	1.3191	0.7412	1.4700
SOD	RC2	0.5643	0.7516	0.5144	0.5678	0.8349	0.6515
	RMSEC	31.4253	23.5164	34.5800	30.6574	15.2350	27.0500
	RMSEV	31.5300	23.6412	32.2543	30.6574	15.7850	28.9300
	RP2	0.6599	0.6537	0.5837	0.5067	0.5829	0.5866
	RMSEP	19.1623	20.9562	31.0400	32.1085	30.0600	29.3200
	RPD	1.6486	1.6920	1.5262	0.9850	1.5174	1.5730
LTRI	RC2	0.9203	0.9437	0.8722	0.8387	0.9250	0.9570
	RMSEC	0.2898	0.2569	0.3250	0.4077	0.2850	0.2470
	RMSEV	0.2948	0.2619	0.3627	0.4127	0.2870	0.2490
	RP2	0.8566	0.8559	0.8592	0.8427	0.7871	0.8902
	RMSEP	0.3752	0.3606	0.3401	0.3887	0.4394	0.3250
	RPD	2.6439	2.5490	2.6602	2.4270	2.1395	2.9036

CARS

SVM	RF	PLS	BP	LSTM	CNN	SVM
0.9340	0.9429	0.9218	0.9210	0.9370	0.9204	0.9240
2.0920	2.0243	2.3400	2.3407	2.0940	2.1402	2.2480
2.1180	2.0243	2.3282	2.4035	2.1033	2.3402	2.3180
0.8602	0.8308	0.8564	0.8618	0.8054	0.8464	0.8579
2.9879	3.2470	3.0200	2.9005	3.9551	3.1720	3.0024
2.6489	2.3442	2.6055	2.7106	2.2820	2.5850	2.6251
0.8830	0.9017	0.7116	0.7230	0.8750	0.8194	0.8021
1.8093	1.8737	2.8700	2.7765	1.9182	2.1300	2.3493
1.8093	1.8737	2.9026	2.7765	1.9260	2.0100	2.3493
0.7510	0.7501	0.7636	0.6128	0.7958	0.7419	0.7640
2.6161	2.5483	2.4300	3.1912	2.3447	2.5800	2.5767
1.9707	1.5723	1.9210	1.5873	2.1654	1.5600	2.0483
0.9044	0.8429	0.6674	0.6610	0.8670	0.8493	0.8220
1.7484	2.3507	2.3200	3.3003	2.1990	2.3108	2.3853
1.7484	2.3507	3.2569	3.4130	2.2140	2.3108	2.3913
0.7287	0.6507	0.6349	0.6793	0.6521	0.6779	0.6209
3.2108	3.4009	0.7968	3.2858	3.5608	3.3729	3.6537
1.8824	1.3190	1.4193	1.7664	1.6091	1.7850	1.5044
0.7127	0.8376	0.6928	0.7194	0.7120	0.7536	0.7632
2.7217	2.1021	2.8100	2.6804	2.6102	2.7102	2.4612
2.7217	2.1021	2.8029	2.6804	2.6243	2.4000	2.4612
0.6796	0.6239	0.6672	0.7044	0.6835	0.6401	0.7396
3.0088	3.0302	2,92	2.7421	2.8413	2.9613	2.5711
1.7225	1.3783	1.5367	1.7959	1.6550	1.4550	1.8551
0.7212	0.8487	0.5178	0.5195	0.5610	0.7395	0.5635
1.5907	1.3925	2.3400	2.3200	2.1960	1.3360	1.9762
1.5907	1.3925	2.0738	2.0860	2.2360	1.0300	2.0762
0.4773	0.5196	0.4052	0.2878	0.2290	0.4923	0.4202
2.1312	1.9806	2.2800	2.5611	2.7534	2.1350	2.2530
1.2008	0.9205	0.9650	1.1192	0.9291	1.3500	1.0990
0.5793	0.7528	0.5800	0.6275	0.7230	0.7051	0.7208
30.7089	23.6177	30.8300	28.4029	24.3631	26.2500	25.3451
31.3740	23.6177	29.9873	28.4029	24.4317	27.4500	25.3451
0.6820	0.6402	0.6172	0.6030	0.5754	0.5411	0.6983
18.7720	22.3446	23.8700	26.9577	26.7523	30.3200	19.4018
1.7312	1.6583	1.6119	1.1732	1.5508	1.3730	1.8275
0.9325	0.9405	0.8623	0.7496	0.9070	0.9126	0.9695
0.2668	0.2937	0.3360	0.5163	0.2821	0.2713	0.1775
0.2668	0.2937	0.3765	0.5612	0.2732	0.2722	0.1725
0.8548	0.8199	0.8369	0.7767	0.7963	0.7700	0.7795
0.3756	0.4029	0.3838	0.4468	0.4653	0.4892	0.4734
2.6387	2.0483	2.4727	2.1364	2.1862	1.9861	2.1244

SPA					NO		
**RF**	**PLS**	**BP**	**LSTM**	**CNN**	**SVM**	**RF**	**PLS**
0.9541	0.9305	0.9037	0.9376	0.9520	0.9299	0.9625	0.9386
1.7926	1.9250	2.5784	1.8366	1.7402	2.1542	1.6361	2.0111
1.7926	2.1954	2.6184	1.8620	1.6400	2.2093	1.725	2.3411
0.8328	0.8645	0.8206	0.8011	0.8666	0.8593	0.8397	0.8549
3.2443	3.1025	3.3277	3.6432	3.1072	3.0114	3.1699	3.085
2.3894	2.7234	2.3626	2.1549	2.7855	2.6409	2.4381	2.6081
0.8867	0.6278	0.6841	0.8620	0.8019	0.8149	0.929	0.7128
1.9658	3.5600	2.9768	2.0073	2.4073	2.2672	1.5375	2.8233
1.9658	3.2145	2.9768	2.1336	2.4070	2.3252	1.634	2.934
0.7673	0.6545	0.5956	0.7481	0.7307	0.7466	0.784	0.7416
2.4544	2.9800	3.2025	2.9216	3.0216	2.634	2.3322	2.5678
1.6395	1.5650	1.5817	1.9193	1.8130	1.9459	1.8877	1.8212
0.8411	0.5984	0.6236	0.7790	0.7393	0.9106	0.901	0.7195
2.4402	3.7800	3.4662	2.7940	3.1080	1.6928	1.9195	2.9911
2.4402	3.5788	3.4662	2.8370	3.1230	1.7361	2.035	3.241
0.6517	0.6577	0.7438	0.6691	0.6254	0.7682	0.6871	0.753
3.4433	3.4200	2.9600	3.6302	3.6729	2.8344	3.2499	2.8839
1.2001	1.3934	1.9608	1.2260	1.5850	1.9577	1.3464	1.822
0.8576	0.6990	0.7622	0.8020	0.7563	0.7784	0.9093	0.679
2.0631	2.8700	2.4793	2.3350	2.5102	2.381	1.6249	2.8654
2.0631	2.7744	2.4793	2.3250	2.6700	2.4419	1.724	2.765
0.7317	0.6375	0.7146	0.6005	0.6580	0.7631	0.6778	0.6655
2.6005	3.1200	2.7094	3.8921	3.5960	2.5084	2.8283	2.8963
1.4789	1.4396	1.8176	1.5751	1.4550	1.9509	1.5112	1.4891
0.7929	0.5142	0.5796	0.4620	0.7850	0.7226	0.8992	0.644
1.5309	2.3800	2.0174	2.7170	1.5360	1.604	1.077	1.7819
1.5309	2.0814	2.0174	2.8650	1.4036	1.6451	1.131	1.872
0.3216	0.3991	0.4087	0.1142	0.5328	0.4995	0.5708	0.5581
2.3573	2.2800	2.3470	3.6770	1.8300	2.0287	1.8684	2.0273
0.8121	0.9700	1.2213	0.8111	1.2400	1.136	1.1351	1.368
0.7829	0.4997	0.5728	0.5632	0.7605	0.7216	0.8024	0.5119
22.3655	35.6700	30.3479	31.4232	24.5700	25.4014	21.5012	32.3593
22.3655	32.7264	31.0491	31.6782	23.7900	26.0516	23.61	33.65
0.6251	0.5777	0.6574	0.2839	0.5825	0.7002	0.6596	0.5693
22.8035	30.7900	22.1418	36.1957	27.3200	19.176	21.3304	21.91
1.6330	1.4813	1.4284	1.1490	1.5236	1.8149	1.7206	1.4297
0.9251	0.8422	0.8585	0.8210	0.8710	0.9293	0.9439	0.8972
0.2900	0.3470	0.3829	0.3910	0.3213	0.2722	0.257	0.3252
0.3100	0.4030	0.3293	0.3872	0.3167	0.2791	0.256	0.3321
0.8398	0.8483	0.8688	0.7620	0.7273	0.8603	0.854	0.8577
0.3838	0.3462	0.3579	0.4573	0.5422	0.3733	0.3628	0.3893
2.4523	2.5564	2.6667	2.0697	1.8815	2.6861	2.5337	2.6537

NE

BP	LSTM	CNN
0.7976	0.927	0.9071
4.1689	2.134	2.4809
4.2689	2.25	2.509
0.7361	0.8215	0.8334
4.7114	3.451	3.2073
1.6687	2.36	2.3719
0.6912	0.862	0.7799
3.1461	1.243	2.5092
2.8613	1.3318	2.792
0.7231	0.6281	0.6908
2.8613	3.15	2.8247
1.61	1.6318	1.6471
0.6513	0.7983	0.6407
3.3818	2.404	3.3961
3.518	2.381	3.61
0.7036	0.4901	0.6371
3.1397	4.671	3.4731
1.8486	1.2034	1.2808
0.7437	0.7923	0.7338
2.5824	2.4751	2.611
2.5924	2.652	2.831
0.7216	0.5917	0.7132
2.607	3.436	2.7027
1.889	1.5338	1.6716
0.1203	0.5984	0.7399
2.9373	2.1277	1.531
3.3373	2.356	1.631
0.1618	0.2527	0.5262
2.7123	3.012	2.0106
1.0568	0.9817	1.2467
0.6994	0.862	0.6679
25.6446	10.6394	27.0288
26.446	11.25	28.0288
0.6204	0.6873	0.5527
25.7412	19.872	24.5525
1.2287	1.702	1.452
0.9217	0.9531	0.8904
0.2879	0.1022	0.3376
0.2979	0.1106	0.3562
0.7914	0.8059	0.8137
0.4777	0.4367	0.4262
1.9983	2.0862	2.3146
